# 2-(2-Chloro­phen­yl)-2-oxo-*N*-phenyl­acetamide

**DOI:** 10.1107/S1600536811044886

**Published:** 2011-11-02

**Authors:** Jing Dai, Jin-Long Wu

**Affiliations:** a Laboratory of Asymmetric Catalysis and Synthesis, Department of Chemistry, Zhejiang University, Hangzhou, Zhejiang 310027, People’s Republic of China

## Abstract

In the title compound, C_14_H_10_ClNO_2_, the dihedral angle between the two rings is 59.4 (2)°. The two carbonyl groups are oriented almost anti­periplanar to each other, with a torsion angle of −160.43 (2)°. In the crystal, mol­ecules are linked into inversion dimers by pairs of N—H⋯O hydrogen bonds.

## Related literature

The crystal structure of the title compound was determined within a project on the synthesis of new  phenylacetamides, see: Li & Wu (2010 ▶).
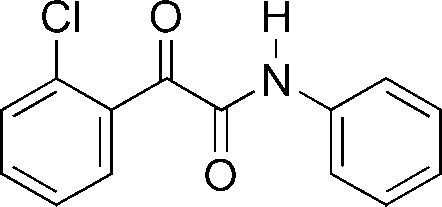


## Experimental

### 

#### Crystal data

C_14_H_10_ClNO_2_
*M* *_r_* = 259.68
Monoclinic, 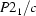

*a* = 11.3513 (11) Å
*b* = 10.4585 (8) Å
*c* = 10.2944 (10) Å
β = 100.954 (10)°
*V* = 1199.86 (19) Å^3^
*Z* = 4
Mo *K*α radiation
μ = 0.31 mm^−1^
*T* = 293 K
0.48 × 0.39 × 0.25 mm

#### Data collection

Oxford Diffraction Xcalibur Atlas Gemini ultra diffractometer
Absorption correction: multi-scan (*CrysAlis PRO*; Oxford Diffraction, 2009 ▶) *T*_min_ = 0.860, *T*_max_ = 0.928
5282 measured reflections
2201 independent reflections
1562 reflections with *I* > 2σ(*I*)
*R*_int_ = 0.021

#### Refinement

*R*[*F* ^2^ > 2σ(*F* ^2^)] = 0.034
*wR*(*F* ^2^) = 0.095
*S* = 1.01
2201 reflections
163 parameters
H-atom parameters constrained
Δρ_max_ = 0.14 e Å^−3^
Δρ_min_ = −0.19 e Å^−3^

### 

Data collection: *CrysAlis PRO* (Oxford Diffraction, 2009 ▶); cell refinement: *CrysAlis PRO*; data reduction: *CrysAlis PRO*; program(s) used to solve structure: *SHELXS97* (Sheldrick, 2008 ▶); program(s) used to refine structure: *SHELXL97* (Sheldrick, 2008 ▶); molecular graphics: *OLEX2* (Dolomanov *et al.*, 2009 ▶); software used to prepare material for publication: *OLEX2*.

## Supplementary Material

Crystal structure: contains datablock(s) I, global. DOI: 10.1107/S1600536811044886/nc2246sup1.cif

Structure factors: contains datablock(s) I. DOI: 10.1107/S1600536811044886/nc2246Isup2.hkl

Supplementary material file. DOI: 10.1107/S1600536811044886/nc2246Isup3.cml

Additional supplementary materials:  crystallographic information; 3D view; checkCIF report

## Figures and Tables

**Table 1:** Hydrogen-bond geometry (Å, °)

*D*—H⋯*A*	*D*—H	H⋯*A*	*D*⋯*A*	*D*—H⋯*A*
N—H⋯O1^i^	0.86	2.52	3.241 (4)	141

Symmetry code: (i) 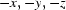.

**Fig. 1.**
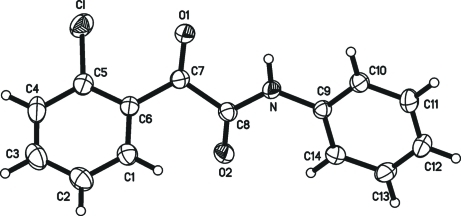
The molecular structure of the title compound with the atom numbering scheme. Displacement ellipsoids are drawn at 40% probability level. H atoms are presented as a small spheres of arbitrary radius.

**Fig. 2.**
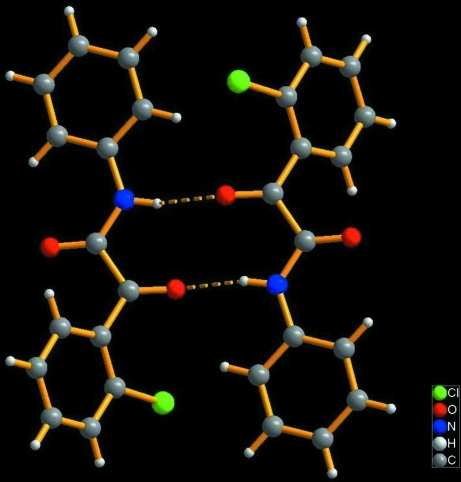
The dimer of the title compound linked by two N—H···O hydrogen bonds (dotted lines).

## supplementary crystallographic information

### Experimental

A solution of 2-chloroacetophenone (1.0 g, 6.5 mmol) and SeO_2_ (1.94 g, 16.8 mmol) in 10 ml of freshly distilled pyridine was heated to 383 K. The reaction mixture was gradually cooled down to 363 K over 1 h and was kept at thistemperature for additional 4 h. The solution was concentrated using a rotary evaporator until a small amount of liquid was present. The black selenium was rinsed several times with ethyl acetate. The combined organic layers were acidified with 10 ml of 0.1 *M* HC1 and the aqueous layer was extracted three times with ethyl acetate. The organic layers were combined and extracted several times with saturated aqueous NaHC0_3_. The aqueous layers were combined, brought to pH 1 with conc. HCl and extracted three times with ethyl acetate. The final organic layers were dried over anhydrous Na_2_SO_4_ and concentrated, producing (2-chlorophenyl)glyoxylic acid in 85% yield (1.02 g) as a solid.

Into a suspension of (2-chlorophenyl)glyoxylic acid (250 mg, 1.36 mmol) and aniline (116 mg, 1.25 mmol) in methylene chloride (8 ml), *N*,*N*'-dicyclohexylcarbodiimide (DCC) (280 mg, 1.36 mmol) and 4-(dimethylamino)pyridine (DMAP) (33 mg, 0.27 mmol) was added respectively at room temperature and continuted stirring for 8 h. The reaction mixture was filtered and the filtrate was concentrated under reduced pressure, the residue was purified by colum chromatography (silica gel, 30% of ethyl acetate in hexane) to afford the title compound in 72% yield (254 mg) as a white solid, m.p. 349–351 K, ^1^H NMR (400 MHz, CDCl_3_) /d 8.79 (brs, 1H), 7.74 (d, J = 7.6 Hz, 1H), 7.70 (d, J = 8.0 Hz, 2H), 7.53–7.47 (m, 2H), 7.40 (t, J = 8.0 Hz, 3H), 7.21 (t, J = 7.6 Hz, 1H). Single crystals suitable for X-ray diffraction of the title compound were grown in a micture of ethyl acetate and hexane.

### Refinement

The H atoms were placed in calculated positions with C—H = 0.93 Å and refined isotropic with *U*_iso_(H) =1.2*U*_eq_ of the carrier atom using a riding model.

### Crystal data

**Table:**

C_14_H_10_ClNO_2_	*F*(000) = 536
*M**_r_* = 259.68	*D*_x_ = 1.438 Mg m^−^^3^
Monoclinic, *P*2_1_/*c*	Mo *K*α radiation, λ = 0.71073 Å
Hall symbol: -P 2ybc	Cell parameters from 1968 reflections
*a* = 11.3513 (11) Å	θ = 3.5–29.2°
*b* = 10.4585 (8) Å	µ = 0.31 mm^−^^1^
*c* = 10.2944 (10) Å	*T* = 293 K
β = 100.954 (10)°	Block, yellow
*V* = 1199.86 (19) Å^3^	0.48 × 0.39 × 0.25 mm
*Z* = 4	

### Data collection

**Table:**

Oxford Diffraction Xcalibur Atlas Gemini ultra diffractometer	2201 independent reflections
Radiation source: fine-focus sealed tube	1562 reflections with *I* > 2σ(*I*)
graphite	*R*_int_ = 0.021
Detector resolution: 10.3592 pixels mm^-1^	θ_max_ = 25.4°, θ_min_ = 3.6°
ω scans	*h* = −13→13
Absorption correction: multi-scan (*CrysAlis PRO*; Oxford Diffraction, 2009)	*k* = −11→12
*T*_min_ = 0.860, *T*_max_ = 0.928	*l* = −12→7
5282 measured reflections	

### Refinement

**Table:**

Refinement on *F*^2^	Primary atom site location: structure-invariant direct methods
Least-squares matrix: full	Secondary atom site location: difference Fourier map
*R*[*F*^2^ > 2σ(*F*^2^)] = 0.034	Hydrogen site location: inferred from neighbouring sites
*wR*(*F*^2^) = 0.095	H-atom parameters constrained
*S* = 1.01	*w* = 1/[σ^2^(*F*_o_^2^) + (0.0552*P*)^2^] where *P* = (*F*_o_^2^ + 2*F*_c_^2^)/3
2201 reflections	(Δ/σ)_max_ < 0.001
163 parameters	Δρ_max_ = 0.14 e Å^−^^3^
0 restraints	Δρ_min_ = −0.19 e Å^−^^3^

### Special details

**Table:**

Geometry. All e.s.d.'s (except the e.s.d. in the dihedral angle between two l.s. planes) are estimated using the full covariance matrix. The cell e.s.d.'s are taken into account individually in the estimation of e.s.d.'s in distances, angles and torsion angles; correlations between e.s.d.'s in cell parameters are only used when they are defined by crystal symmetry. An approximate (isotropic) treatment of cell e.s.d.'s is used for estimating e.s.d.'s involving l.s. planes.
Refinement. Refinement of *F*^2^ against ALL reflections. The weighted *R*-factor *wR* and goodness of fit *S* are based on *F*^2^, conventional *R*-factors *R* are based on *F*, with *F* set to zero for negative *F*^2^. The threshold expression of *F*^2^ > σ(*F*^2^) is used only for calculating *R*-factors(gt) *etc*. and is not relevant to the choice of reflections for refinement. *R*-factors based on *F*^2^ are statistically about twice as large as those based on *F*, and *R*- factors based on ALL data will be even larger.

### Fractional atomic coordinates and isotropic or equivalent isotropic displacement parameters (Å^2^)

**Table:**

	*x*	*y*	*z*	*U*_iso_*/*U*_eq_	
Cl	0.39169 (4)	0.11424 (5)	0.07892 (5)	0.0671 (2)	
O1	0.12583 (11)	0.05846 (11)	−0.01363 (12)	0.0534 (4)	
O2	0.04597 (11)	0.32465 (12)	−0.20894 (12)	0.0556 (4)	
N	−0.05486 (12)	0.13663 (13)	−0.20367 (13)	0.0438 (4)	
H	−0.0482	0.0637	−0.1643	0.053*	
C1	0.13889 (17)	0.38201 (17)	0.07314 (17)	0.0505 (5)	
H1	0.0609	0.3986	0.0295	0.061*	
C2	0.1960 (2)	0.46949 (19)	0.16282 (18)	0.0627 (5)	
H2	0.1563	0.5432	0.1811	0.075*	
C3	0.3117 (2)	0.4475 (2)	0.2250 (2)	0.0697 (6)	
H3	0.3508	0.5067	0.2856	0.084*	
C4	0.37045 (18)	0.3389 (2)	0.19889 (19)	0.0637 (6)	
H4	0.4495	0.3254	0.2407	0.076*	
C5	0.31215 (15)	0.24881 (17)	0.10994 (18)	0.0483 (5)	
C6	0.19425 (14)	0.26908 (16)	0.04560 (16)	0.0409 (4)	
C7	0.12143 (13)	0.17184 (16)	−0.03847 (16)	0.0404 (4)	
C8	0.03285 (14)	0.22088 (16)	−0.16030 (16)	0.0406 (4)	
C9	−0.15676 (14)	0.15162 (15)	−0.30543 (16)	0.0386 (4)	
C10	−0.24427 (14)	0.05916 (17)	−0.31498 (16)	0.0449 (4)	
H10	−0.2331	−0.0109	−0.2583	0.054*	
C11	−0.34801 (16)	0.07007 (19)	−0.40792 (19)	0.0572 (5)	
H11	−0.4074	0.0080	−0.4135	0.069*	
C12	−0.36407 (17)	0.1729 (2)	−0.49283 (19)	0.0609 (5)	
H12	−0.4345	0.1806	−0.5553	0.073*	
C13	−0.27627 (18)	0.26376 (18)	−0.48528 (18)	0.0567 (5)	
H13	−0.2874	0.3326	−0.5436	0.068*	
C14	−0.17155 (16)	0.25492 (16)	−0.39263 (17)	0.0480 (5)	
H14	−0.1120	0.3166	−0.3884	0.058*	

### Atomic displacement parameters (Å^2^)

**Table:**

	*U*^11^	*U*^22^	*U*^33^	*U*^12^	*U*^13^	*U*^23^
Cl	0.0447 (3)	0.0726 (4)	0.0818 (4)	0.0070 (2)	0.0062 (2)	0.0104 (3)
O1	0.0500 (7)	0.0414 (7)	0.0634 (8)	−0.0033 (6)	−0.0028 (6)	0.0056 (6)
O2	0.0601 (8)	0.0440 (8)	0.0578 (8)	−0.0130 (6)	−0.0017 (6)	0.0081 (6)
N	0.0394 (8)	0.0385 (8)	0.0499 (9)	−0.0037 (6)	−0.0002 (7)	0.0092 (6)
C1	0.0499 (11)	0.0514 (11)	0.0501 (11)	−0.0042 (9)	0.0089 (9)	−0.0006 (9)
C2	0.0786 (15)	0.0572 (12)	0.0541 (12)	−0.0067 (11)	0.0174 (11)	−0.0115 (10)
C3	0.0906 (17)	0.0681 (15)	0.0469 (11)	−0.0216 (13)	0.0042 (11)	−0.0083 (10)
C4	0.0551 (12)	0.0788 (15)	0.0501 (11)	−0.0204 (11)	−0.0075 (9)	0.0080 (11)
C5	0.0444 (10)	0.0534 (11)	0.0467 (10)	−0.0062 (8)	0.0077 (8)	0.0080 (8)
C6	0.0405 (9)	0.0438 (10)	0.0384 (9)	−0.0047 (8)	0.0074 (7)	0.0050 (7)
C7	0.0352 (9)	0.0402 (10)	0.0466 (10)	−0.0006 (7)	0.0100 (7)	0.0030 (8)
C8	0.0385 (9)	0.0397 (10)	0.0441 (10)	−0.0034 (8)	0.0087 (7)	0.0006 (8)
C9	0.0367 (9)	0.0397 (9)	0.0390 (9)	0.0038 (7)	0.0063 (7)	−0.0013 (7)
C10	0.0412 (10)	0.0442 (10)	0.0485 (10)	−0.0028 (8)	0.0070 (8)	0.0030 (8)
C11	0.0412 (10)	0.0627 (13)	0.0632 (12)	−0.0080 (9)	−0.0013 (9)	−0.0011 (10)
C12	0.0484 (11)	0.0669 (13)	0.0597 (12)	0.0075 (10)	−0.0091 (9)	−0.0009 (10)
C13	0.0666 (13)	0.0495 (11)	0.0496 (11)	0.0108 (10)	−0.0002 (10)	0.0076 (8)
C14	0.0524 (11)	0.0401 (10)	0.0497 (11)	−0.0034 (8)	0.0053 (9)	0.0023 (8)

### Geometric parameters (Å, °)

**Table:**

Cl—C5	1.7342 (19)	C5—C6	1.392 (2)
O1—C7	1.2120 (19)	C6—C7	1.481 (2)
O2—C8	1.2161 (19)	C7—C8	1.539 (2)
N—C8	1.341 (2)	C9—C10	1.376 (2)
N—C9	1.414 (2)	C9—C14	1.394 (2)
N—H	0.8600	C10—C11	1.374 (2)
C1—C2	1.372 (3)	C10—H10	0.9300
C1—C6	1.392 (2)	C11—C12	1.376 (3)
C1—H1	0.9300	C11—H11	0.9300
C2—C3	1.366 (3)	C12—C13	1.369 (3)
C2—H2	0.9300	C12—H12	0.9300
C3—C4	1.370 (3)	C13—C14	1.378 (2)
C3—H3	0.9300	C13—H13	0.9300
C4—C5	1.391 (3)	C14—H14	0.9300
C4—H4	0.9300		
			
C8—N—C9	128.68 (14)	C6—C7—C8	116.93 (14)
C8—N—H	115.7	O2—C8—N	126.20 (15)
C9—N—H	115.7	O2—C8—C7	121.39 (14)
C2—C1—C6	121.93 (18)	N—C8—C7	112.41 (14)
C2—C1—H1	119.0	C10—C9—C14	120.10 (15)
C6—C1—H1	119.0	C10—C9—N	116.92 (14)
C3—C2—C1	119.5 (2)	C14—C9—N	122.98 (15)
C3—C2—H2	120.3	C11—C10—C9	120.19 (17)
C1—C2—H2	120.3	C11—C10—H10	119.9
C2—C3—C4	120.56 (18)	C9—C10—H10	119.9
C2—C3—H3	119.7	C10—C11—C12	119.99 (18)
C4—C3—H3	119.7	C10—C11—H11	120.0
C3—C4—C5	120.16 (19)	C12—C11—H11	120.0
C3—C4—H4	119.9	C13—C12—C11	119.97 (17)
C5—C4—H4	119.9	C13—C12—H12	120.0
C4—C5—C6	120.28 (18)	C11—C12—H12	120.0
C4—C5—Cl	118.15 (15)	C12—C13—C14	121.04 (17)
C6—C5—Cl	121.54 (14)	C12—C13—H13	119.5
C5—C6—C1	117.56 (15)	C14—C13—H13	119.5
C5—C6—C7	123.55 (16)	C13—C14—C9	118.68 (16)
C1—C6—C7	118.56 (14)	C13—C14—H14	120.7
O1—C7—C6	123.52 (14)	C9—C14—H14	120.7
O1—C7—C8	119.50 (14)		

### Hydrogen-bond geometry (Å, °)

**Table:**

*D*—H···*A*	*D*—H	H···*A*	*D*···*A*	*D*—H···*A*
N—H···O1^i^	0.86	2.52	3.241 (4)	141.

Symmetry codes: (i) −*x*, −*y*, −*z*.
